# Magnetically Tunable Hydrogel for Biofilm Control

**DOI:** 10.1021/acsabm.5c00409

**Published:** 2025-05-19

**Authors:** Ruojiao Sun, Manasi S. Gangan, Qiming Wang, James Q. Boedicker, Andrea M. Armani

**Affiliations:** a Mork Family Department of Chemical Engineering and Materials Science, 5116 University of Southern California, Los Angeles, California 90089, United States; b Department of Physics and Astronomy, 115098 University of Southern California, Los Angeles, California 90089, United States; c Sonny Astani Department of Civil and Environmental Engineering, 5116 University of Southern California, Los Angeles, California 90089, United States; d Ellison Medical Institute, Los Angeles, California 90064-1016, United States

**Keywords:** biofilm control, magnetic responsive, mechanical properties, nanocomposite substrate, tunable hydrogel

## Abstract

Bacterial biofilm formation contributes to healthcare and energy challenges, and researchers are actively pursuing a range of strategies to restrict the spread of biofilms in an eco-friendly manner. Commonly used approaches in industry rely on physical removal and chemical techniques, frequently targeting mature biofilms. While effective, these methods often face implementation challenges in remote settings and can have off-target environmental impacts. As a result, an alternative strategy is to focus on controlling or limiting the biofilm formation and growth rates with remote stimuli. It has been shown that the mechanotransduction pathway intrinsic to bacteria responds to changes in the storage modulus of the growth surface, modifying the bacteria’s motility and biofilm formation. We developed a material with magnetically tunable mechanical properties by intercalating magnetic nanoparticles into an agar gel matrix and investigated its ability to control Escherichia coli motility and biofilm growth. The initial storage modulus ranges from 0.5 to 2.5 kPa, depending on the material composition. Upon exposure to a 20 mT magnetic field using standard neodymium magnets, the modulus is dynamically and reversibly increased by approximately 30%. As a result of this increase, the expansion rate of the E. coli biofilm is reduced by approximately 40%. The simplicity of the manipulation of its mechanical property not only gives this biomaterial potential to further mechanosensing mechanism research but also proves to be an innovative strategy for remote and eco-conscious restriction of biofilm formation.

## Graphical abstract

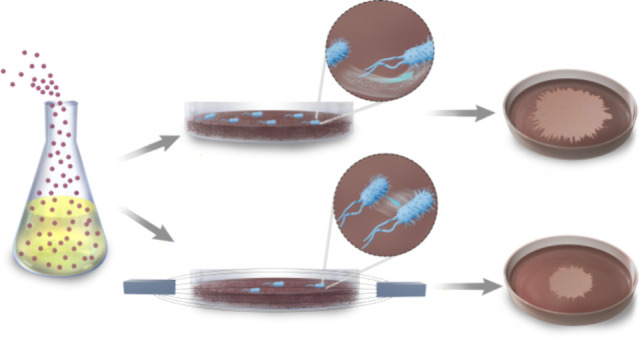

## 1 Introduction

Biofouling has become a daunting challenge with profound impacts on human health, civil infrastructure, and military equipment. For example, increased risks of infection and medical device failures,^1–4^ safety hazards in civilian infrastructure,^5,6^ and corrosion of equipment in a wide range of industries^7–9^ can be attributed to biofouling. Indirect effects are particularly evident in the marine industry, where biofouling increases the drag on vessels, which reduces their energy efficiency.^8,10^ Therefore, preventing biofouling has become necessary across a range of fields.

Prior research on biofouling has suggested it is a multistep process that includes the formation of increasingly complex biological communities. Briefly, after a surface is conditioned with organic matter, a single- or multispecies bacterial biofilm can form. This layer attracts more complex microorganisms, such as fungi and algae, eventually leading to the formation of macrobiological structures, which increase the resistance to complete eradication of biocontaminants from the surface.^11–13^ Therefore, developing strategies to restrict, inhibit, or disrupt the initial stage of biofilm formation before the microorganisms attach provides a path to limit biofouling.

Conventional approaches to reduce biofilm formation include the timely application of antibacterial or harsh chemical agents or biofilm removal using mechanical forces, such as shear flow or external sonics and mechanical vibrations.^14–18^ Additionally, the inhibition of biofilm formation using surfaces resistant to bacterial adhesion has been explored.^19–23^ However, these techniques often require direct interaction, which can be limited in accuracy and scalability, and many of the chemicals have detrimental environmental impacts due to undesired off-target interactions.^24–27^ Furthermore, the long-term efficacy of these approaches remains uncertain, as the presence of even a small population of adherent bacterial cells has the potential to form robust biofilms over time.^28^ As a result, remote manipulation of bacterial behavior has emerged as a potential solution.^29–31^

A unique feature of microbial cells is their ability to sense and adapt to changing mechanical properties of a growth surface or substratum.^32–34^ Past work has shown that both Gram-positive and Gram-negative bacterial cells can dynamically modify their cell wall stiffness to align with a stiffer surface.^35,36^ This increase has been associated with higher levels of cyclic diguanylate (cyclic-di-GMP), which plays a critical role in regulating proteins, enzymes, and exopolysaccharides in bacterial signaling pathways and in the function of flagella, which control bacterial growth and motility.^37,38^ High cyclic-di-GMP levels reduce bacterial motility and the likelihood of detachment, in turn inhibiting biofilm formation and expansion.^39,40^ Thus, the bacterial motility can be modified by changing the mechanical properties of a surface.

In the present work, we leveraged the knowledge of the intrinsic mechanosensory pathways in bacteria to design and demonstrate a material that can modify the bacterial motility by tuning the stiffness of the culture substrate. The material is a nanocomposite composed of agar infused with iron oxide magnetic nanoparticles (MNPs). Upon introduction of a magnetic field, the storage modulus is increased. In addition to fabricating a range of magnetically tunable (magneto-mechanical) hydrogels with varying moduli and characterizing their magnetically actuated mechanical response, we demonstrate the material’s ability to restrict the bacterial motility using motile Escherichia coli MG1655 as a test system. Notably, with the application of a magnetic field, the expansion rate of the E. coli is decreased from 47.5 to 29.2 mm^2^/h.

## 2 Results and Discussion

### 2.1 Sample Preparation

To create the magneto-mechanical gels, iron oxide (Fe_3_O_4_) nanoparticles with an average diameter of approximately 120 nm (Figure S1) were synthesized and were directly mixed into bacteriological agar. A series of magneto-mechanical gels were prepared by covarying agar and nanoparticle concentrations (Figure S2), and each magneto-mechanical gel formulation was plated in triplicate in Petri dishes with a 35 or 60 mm diameter (Figure 1). The mechanical and magneto-mechanical properties of the gel were characterized in 35 mm-diameter plates (Figure 1a), and the bacterial cultures were plated on both control and magneto-mechanical gel plates of 60 mm diameter (Figure 1b).

**Figure 1:**
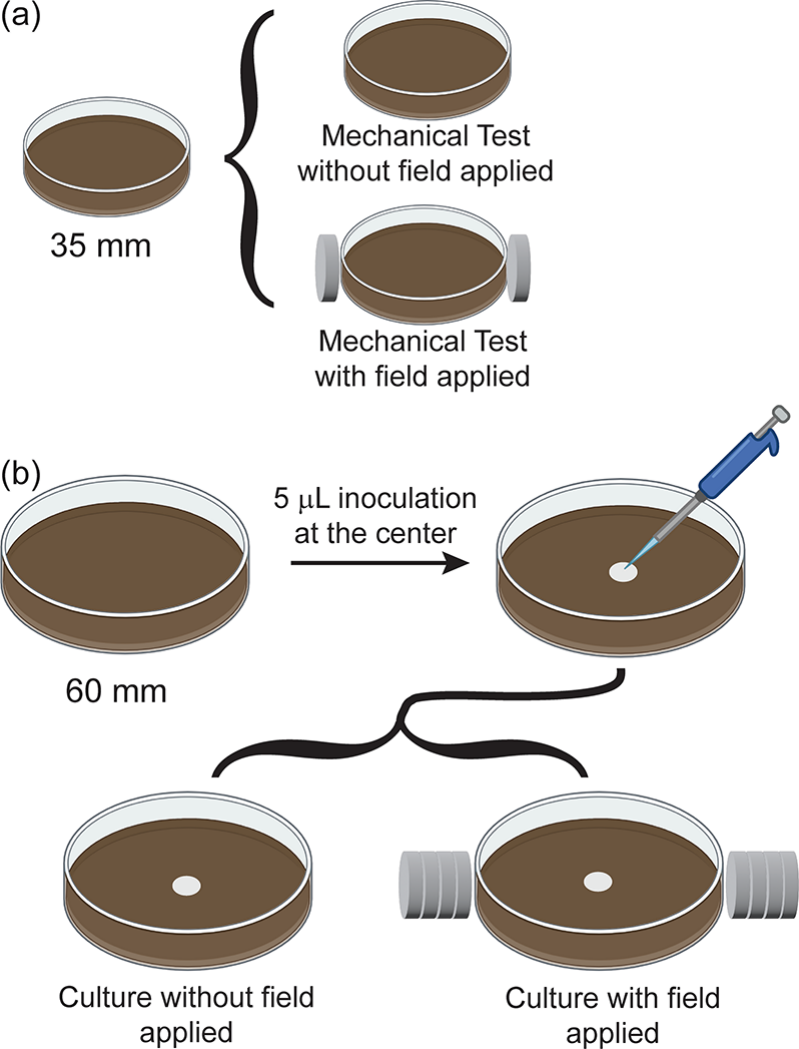
Schematics of the biological and mechanical experiments. (a) The mechanical test samples are prepared in 35 mm Petri dishes, and the magnetic field is generated by placing one magnet on either side of the plate. (b) The biological test samples are prepared in 60 mm Petri dishes, and the magnetic field is generated by placing four stacked magnets on either side of the plate.

The magnetic field is generated by attaching different numbers of magnets to the two-sized plates to ensure the same magnetic field strength at the center of the plates. The effects of the magnetic field on the bacterial behavior were quantified, and the biotoxicity of the material was analyzed. The bacterial growth rate and growth pattern were monitored with an *in situ* imaging system while simultaneously actuating the magnetomechanically responsive hydrogel.

### 2.2 Modeling and Validation of the Magnetic Field Strength Profile

Given that the magnetic field strength directly induces the change in modulus, it is important to experimentally and theoretically analyze the field distribution across the sample. As shown in the modeling results in Figure 2a,c, the magnetic field exhibits a gradient in strength over the entire Petri dish. To account for the larger plate size, additional magnets were used with the 60 mm diameter plates. This approach reduced the impact of the increase in plate size and maintained a consistent field at the center of the plate.

**Figure 2:**
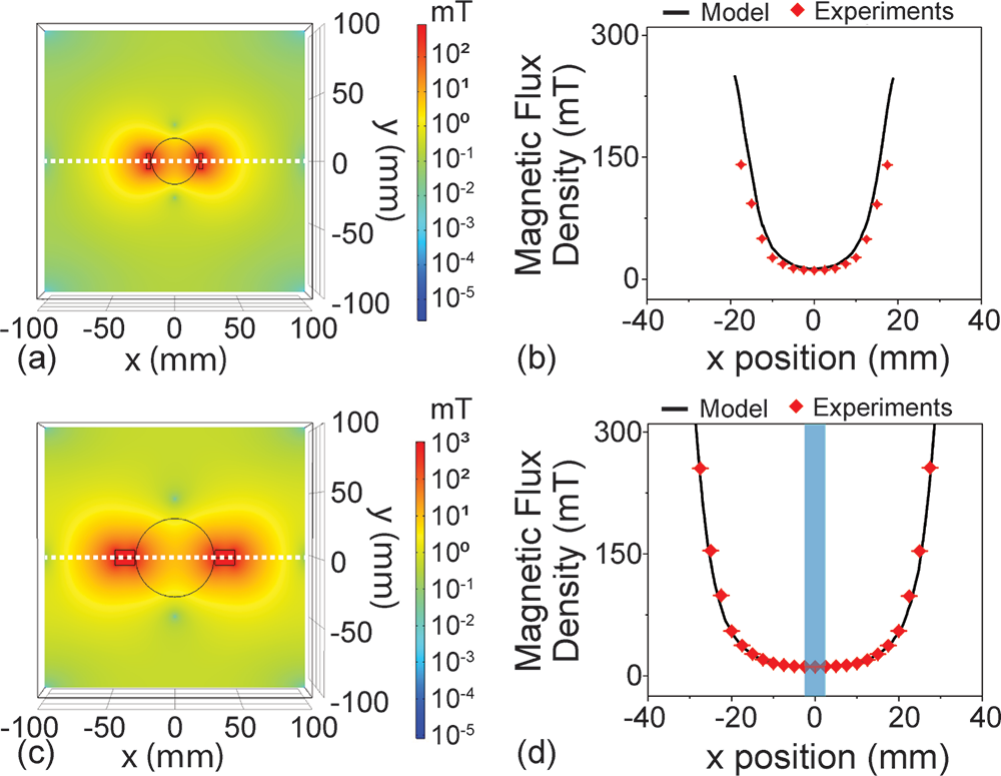
Finite method modeling results. (a) Top view of the distribution of magnetic flux density of the simulated area of the 35 mm plate. The Petri dish boundary and the magnets are indicated by the black line. (b) Distribution of magnetic flux density along the white dotted line at *y* = 0 of the 35 mm plate. (c) Top view of the distribution of magnetic flux density of the simulated area of the 60 mm plate. The Petri dish boundary and the magnets are indicated by the black line. (d) Distribution of magnetic flux density along the white dotted line at *y* = 0 of the 60 mm plate. The shadowed blue region represents the inoculation spot with a diameter of 5 mm.

To visualize the strength of the field more clearly, the magnetic flux density through the center of the Petri dish is plotted as a function of location (Figure 2b,d and Figure S4). Only the region within the Petri dish is included in the plot, and the location of the bacterial inoculation is highlighted in blue. As can be seen, the field is predicted to be uniform near the location of inoculation, and the magnetic flux density values are similar (around 20 mT) for both plates.

Across both diameters, the experimental measurements of the magnetic field are in excellent agreement with the modeling results (Figure 2b,d). Therefore, these modeling results can serve as a calibration of the field strength across the dish in the subsequent bacterial studies.

### 2.3 Stability of Magnetic Nanoparticles within the Gel Matrix

Before assessing the dynamic mechanical properties, the stability of the nanocomposite was investigated over a 24 h time frame. The stability of the MNPs within the matrix was monitored by comparing the diffusive behaviors of the nanoparticles and bromophenol blue dye across the clearly defined boundary region in multimaterial hybrid samples with well-defined regions (Figure 3). The imaging diffusion study was performed for 24 h, and the images were quantified by analyzing the area of colored spots. As can be seen in Figure 3 and Figure S3, the nanoparticles did not noticeably diffuse over this entire time. However, by hour 12, the dye molecules had significantly diffused and the boundaries were beginning to overlap. By hour 24, it was not possible to distinguish the boundaries between the initial spots, and the dye was strongly interacting with the sides of the dish. Therefore, we ended our quantitative comparative analysis at hour 12. Dye diffusion before hour 12 followed classic diffusion behavior (Figure 4). However, the nanoparticles did not display any detectable diffusion during the 24 h test period. This strong confinement indicates that the nanoparticles are trapped in the agar. Additional experimental setup and diffusion analysis results are in the SI.

**Figure 3:**
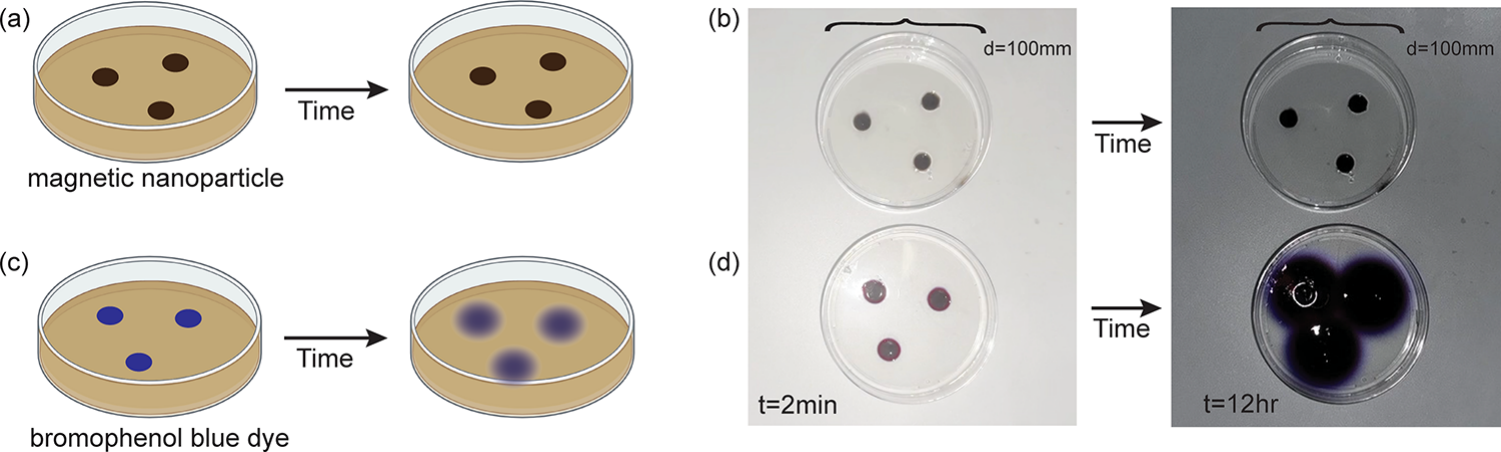
Diffusivity of nanoparticles through agar. (a) Agar gel with magneto-mechanical gel regions in a 100 mm Petri dish. (b) Agar gel with bromophenol blue-dyed gel regions in the 100 mm Petri dish. (c) Two heterogeneous gel systems during a 24 h period. (d) Change of area of the magneto-mechanical gel regions compared to bromophenol blue dye regions at h 12 during the 24 h period.

**Figure 4:**
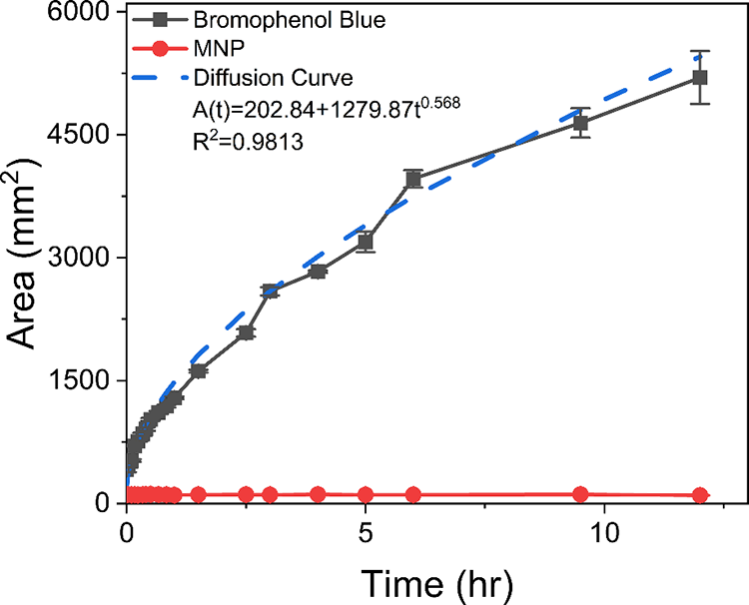
Colored area changes during the first 12 h during the 24 h test period. Magneto-mechanical gel regions do not show diffusion compared to the dye-loaded regions. The diffusion curve of the dye-loaded gel was fitted to a power model, and the diffusion coefficient was found as the derivative of the diffusion curve and a function of time.

In addition, the confinement of the nanoparticle distribution under the influence of a magnetic field was monitored via confocal microscopy imaging at the vertical boundary between the magneto-mechanical gel and the nondoped gel before and after applying a magnetic field for 24 h (Figure 5a). Imaging was performed at different *z*-axis heights (Figure 5b). No detectable diffusion or change in nanoparticle distribution was observed for the magneto-mechanical gel. Additional experimental details are given in the SI.

**Figure 5:**
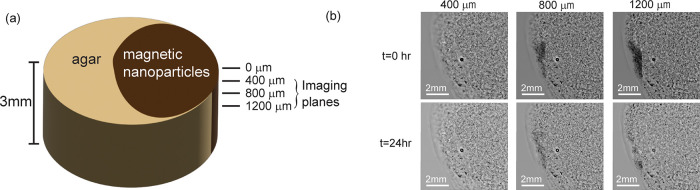
Microscopic images of magneto-mechanical gel under a magnetic field over 24 h. (a) Sample rendering and test setup. (b) Confocal imaging results at three distances from the gel surface before and after 24 h magnetic field application.

### 2.4 Mechanical Analysis of Magneto-Mechanical Gel

During initial mechanical testing of the magneto-mechanical gel, it was observed that the viscoelastic and ultrasoft characteristics of the material resulted in measurement inconsistency due to the presence of the rigid Petri dish. Through an iterative process, we optimized the thickness of the gel to be at least 3.5 mm thick to avoid artifacts from the dish and to obtain reproducible results (Figure S6).

In addition, a series of mechanical tests were completed to quantify the storage moduli of the agar gels, and as expected, they were found to be dependent on the concentration of agar. For agar concentrations from 2 to 8 g/L, the storage moduli ranged from 0.2 to 30 kPa (Figure S7). These findings are in agreement with prior work,^41,42^ and the gels have consistent behaviors during testing. The loss moduli are below 20% of the storage moduli, and minimal residual deformation was observed over the course of the measurements, indicating that the measurement did not cause irreversible sample damage (Figure S7). Based on these results, the test parameters were held fixed for subsequent measurements. Additionally, the inclusion of magnets does not change the results for the gels without nanoparticles, which confirms that the application of the field does not interfere with the instrument’s operation (Figure S8).

Upon introduction of the insoluble MNPs into the gel, the modulus is increased, even in the absence of a magnetic field (Figure 6). Over the range of agar and nanoparticle concentrations studied, the storage modulus could be tuned by nearly an order of magnitude by increasing both agar and nanoparticle concentrations. This increase in modulus, even in the absence of a field, is expected due to the formation of a nanocomposite.^43–45^ However, initial moduli values in excess of 6 kPa can promote bacterial adhesion, restricting bacterial motion.^33,46,47^ Therefore, all subsequent studies using the tunable substrates focused on gels made with 2 to 3 g/L agar and nanoparticle concentrations below 7.5 mg/mL to allow for a clear demonstration of control over cellular motility when a magnetic field is applied.

**Figure 6:**
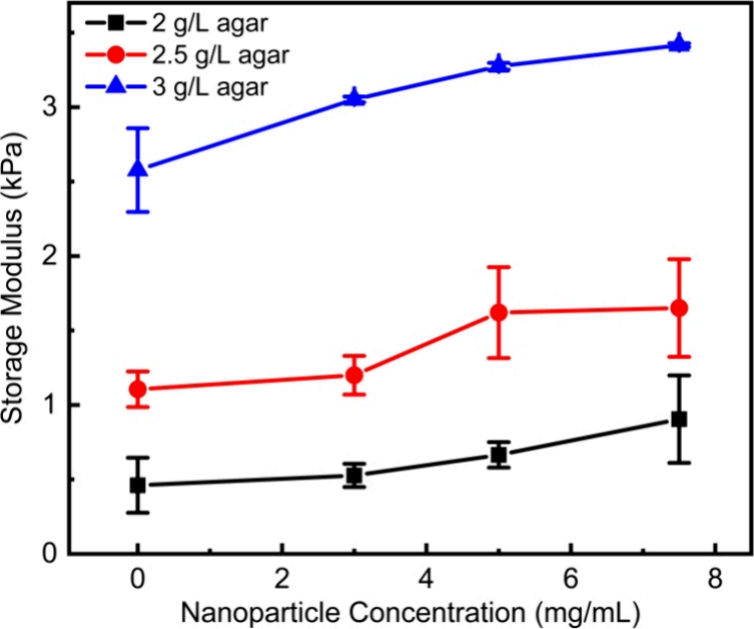
Dynamic mechanical analyzer (DMA) test results for samples at different nanoparticle concentrations. Having nanoparticles in the gel system will stiffen the gel. Each data point consists of three individual measurements from three samples.

To assess the effect of the magnetic field on the storage modulus, a series of samples with covarying nanoparticle concentrations and agar concentrations were prepared in triplicate. Using dynamic mechanical analyzer (DMA), the storage modulus and loss modulus were measured. As can be observed in Figure 7, for all nanoparticle concentrations, the storage modulus of the agar increased with the application of the magnetic field. As in the initial optimization studies, the values for agar hydrogel without nanoparticles compared favorably to prior work.^41,48^

**Figure 7:**
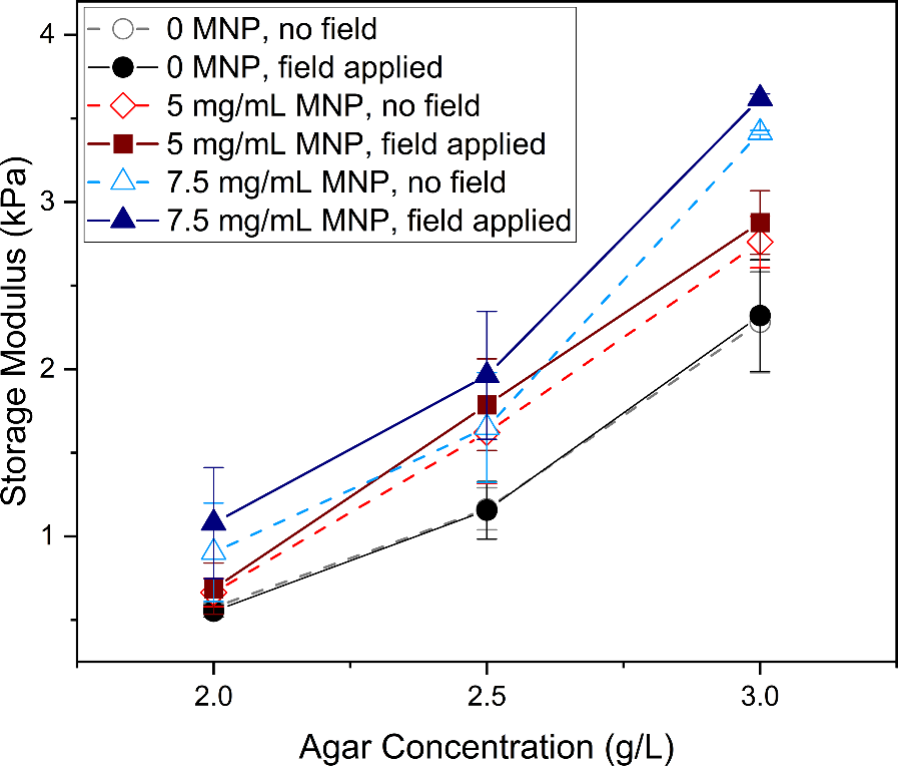
Mechanical test results on magneto-mechanical gels with magnetic field on and off. Nine types of magneto-mechanical gels with three agar concentrations and three MNP concentrations were prepared. Gels with 0 mg/mL MNP showed consistent modulus results when tested with a magnetic field on and off. Gels with 5 and 7.5 mg/mL MNP showed modulus increment when tested with a magnetic field on.

### 2.5 Controlling Bacterial Growth

The motility of this strain of E. coli MG1655 is known for exhibiting a swarming behavior on soft surfaces. Swarming is characterized by the formation of an irregular floral-like pattern that expands outward from the inoculation site.^49^ Based on prior work, it is anticipated that the field strengths used in the present work would not influence the bacterial motility in the absence of a responsive surface.^50^ To verify the hypothesis that the dynamic surface is solely responsible for manipulating the swarming behavior, a series of experiments were performed. These included studying growth dynamics on the magnetically responsive surfaces as well as bacteria grown in normal agar gels.

First, to ensure the application of magnetic fields would not interfere with bacterial growth, a set of plates with 2.5 g/L agar gel were prepared without adding MNPs. A schematic of the bacterial seeding protocol and bacterial swarming behavior during biological test is illustrated in Figure 8a. During incubation, the experimental plates were exposed to the magnetic field, and the control set of plates was placed under normal conditions. Images were acquired every hour, and representative images for two time points, h 8 (when bacterial colony started to become visible) and h 23 (end of incubation), for each condition are shown in Figure 8b. As shown in the images, bacteria did not show noticeable changes in colony area in the presence of magnetic fields. To compare growth rates quantitatively, colonies were imaged during the culture period, and colony areas were calculated using ImageJ (Figure 8d). The rate of the bacterial colony expansion was then calculated and is shown in Figure 8e. Specifically for the gels with 0 mg/mL nanoparticles, the expansion rate when cultured under a magnetic field was 64.6 mm^2^/h, while the expansion rate in the absence of a magnetic field was 65.9 mm^2^/h (Figure 8e). Both values were comparable to each other as well as consistent with previous research.^50^ This result indicates that the magnetic field used in this study does not influence the bacterial growth negatively.

**Figure 8:**
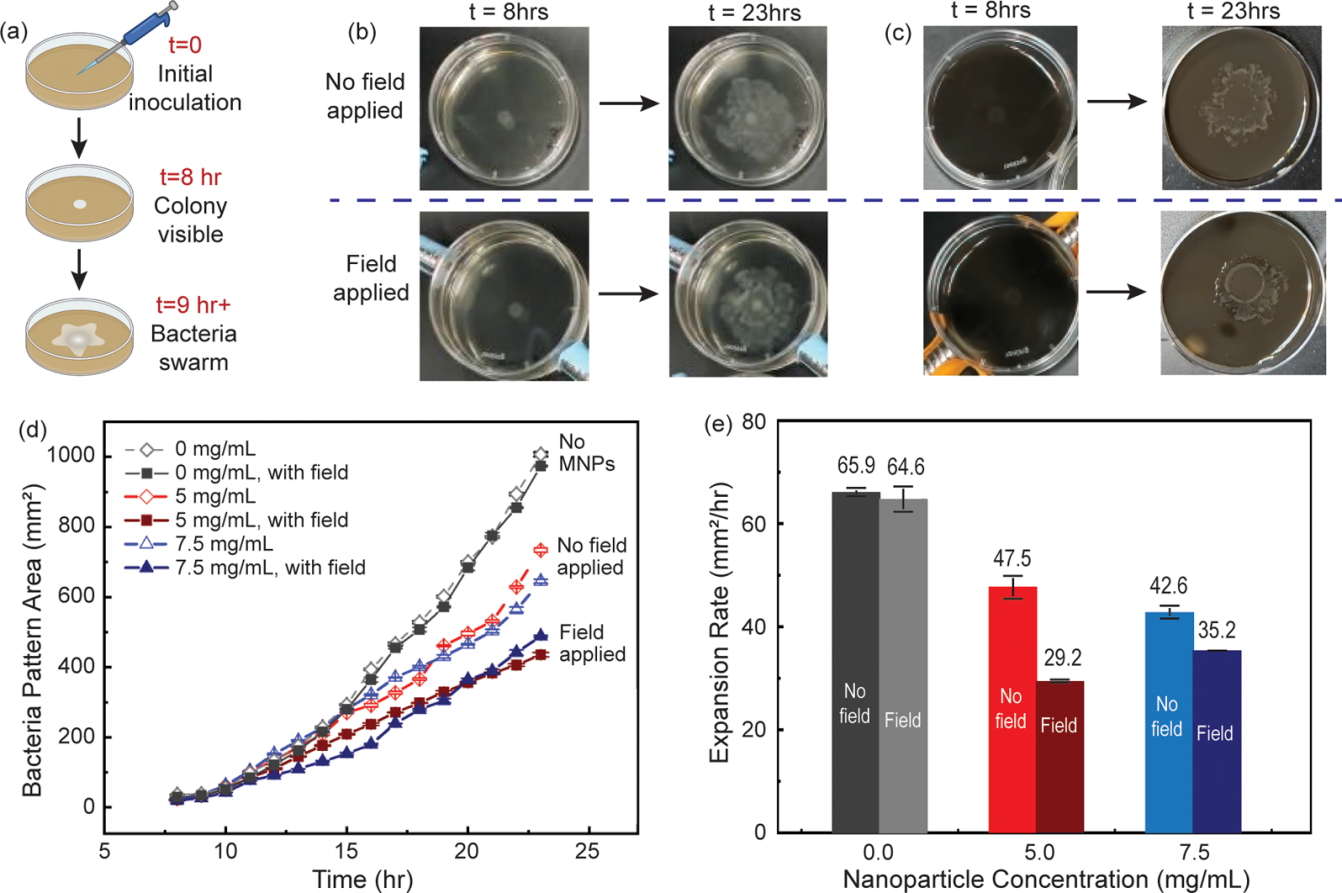
Biological test results on magneto-mechanical gels with magnetic field on and off. (a) Schematic of bacterial seeding protocol and bacterial swarming behavior during the experiment. (b) Bacterial culture on plates with regular agar gels at h 8 and h 23: top row without the magnetic field while culturing, bottom row with the magnetic field while culturing. (c) Bacterial culture on plates with magneto-mechanical gel of 2.5 g/L agar and 5 mg/mL magnetic nanoparticle at h 8 and h 23 at different magnetic field conditions. (d) Quantitative results from ImageJ analysis: The area covered by the bacteria changed over time. Results show averages from three replicate measurements. (e) Expansion rate measurements were averaged from nine samples in three separate sets of experiments. The influence of the magneto-mechanical gel system is significant enough to manipulate bacterial movement.

Based on the results from the mechanical characterization measurements, magneto-mechanical gels with 2.5 g/L agar and 5 or 7.5 mg/mL nanoparticles were prepared in triplicate, and bacteria were seeded at the center of the plates. Two different field conditions were studied. Images were acquired every hour, and representative images for two time points, h 8 and h 23, for the 5 mg/mL samples are shown in Figure 8c. Images for all of the experimental conditions are included in Figure S13 in the SI. As can be qualitatively observed, when the field was applied in combination with the magneto-mechanical gel, the area of the colony decreased. Growth measurements shown in Figure S10 show that nanoparticles themselves do not inhibit cell growth. Therefore, this decreased colony expansion was the result of changes to the mechanical properties of the gel. The quantitative results for both nanoparticle concentrations are shown in Figure 8d. The bacterial motility across the substrate decreased from 47.5 to 29.2 mm^2^/h when the field was applied to the magneto-mechanical gel with a 5 mg/mL nanoparticle concentration. (Figure 8e) This change represents a nearly 40% decrease in expansion from the inoculation site. The bacterial motility across the magneto-mechanical gel with 7.5 mg/mL has less significant change since the nanoparticle loading stiffens the gel and limits the magnetic field effect on gel stiffening. This control over the bacterial motility using the magnetic field is notable, as the substrate material is identical.

## 3 Conclusions

By integrating MNPs within an agar gel matrix, we established an MNP-agar gel composite system capable of modifying the storage modulus of the gel through the application of an external magnetic field. Using this system, biofilm expansion is decreased by over 40% without the use of chemicals with an undesired or off-target environmental impact. It is expected that additional optimization research can further improve this performance or open avenues for new research. For example, modification of the nanoparticle composites could improve durability, and pulsed or oscillating field application would be possible by replacing the neodymium magnets with electromagnets. Furthermore, this dynamically tunable material can also be used in microbiology to simulate more relevant mechanical conditions or changes within one bacterial growth cycle. These studies, combined with an improved understanding of the biochemical changes in the cell such as cyclic-di-GMP levels and genetic expression of flagella, will open doors to a multitude of strategies for stand-off, environmentally friendly control of biofilm formation and biofouling inhibition.^51–53^

## 4 Materials and Methods

### 4.1 Material Synthesis

The magneto-mechanical hydrogel system combines an agar-based hydrogel with iron oxide (Fe_3_O_4_) MNPs. The MNPs were synthesized and purified using a previously developed coprecipitation method, which is detailed in the SI.^54^ The nanoparticle size distribution was measured through dynamic light scattering (Figure S1).

The nanoparticle dispersions were suspended in sterile deionized water (50 mg/mL). The agar-based gel solution was prepared by dissolving and autoclaving bacteriological agar (2 to 8 g/L, Sigma-Aldrich, Missouri, USA), 0.6% (wt/vol) Bacto yeast extract (Gibco, USA), and 2% (wt/vol) Bacto tryptone (Gibco, USA) in distilled water (Figure S2).^55^ An additional 0.5% (wt/vol) of d-glucose (Amresco, USA) was added to the solution after autoclaving.

Next, the iron oxide nanoparticle dispersion was directly mixed into the agar gel solution to create a uniform particle distribution. Throughout this process, the solution was held above 60 °C to remain fluid and allowed for transfer into the plates. Two different sizes of plates were made and allowed to cool down, resulting in the final magneto-mechanical gel (Figure S2). The 60 mm Petri dishes were used for bacterial culture to provide sufficient space for bacterial growth and motility. Due to the limited space inside the mechanical testing equipment, 35 mm Petri dishes were used for mechanical testing. Both mechanical and biological tests were conducted in triplicate from the same gel solution. The same series of tests was performed on at least three occasions, and their results were compared to ensure repeatability. Additional synthesis details are in the SI.

To ensure that the magneto-mechanical gel retained its structural integrity under experimental conditions and to confirm the uniformity of the nanoparticle distribution throughout the matrix, a series of diffusion tests were conducted. Multimaterial hybrid samples that consisted of two discrete regions were designed and fabricated. Each sample had three doped regions surrounded by agar, creating clear interfaces to investigate diffusion or changes in the structure as a result of the magnetic field application. The small molecular dye bromophenol blue was used as a diffusion indicator in the control experiments. The measurements included a pair of 24 h studies with and without the application of a magnetic field and a confocal imaging study to assess the homogeneity of the nanoparticles throughout the matrix. The experimental details are in the SI.

For initial optimization investigations, a series of magneto-mechanical gels were prepared by covarying the agar concentration and the MNP concentration. The agar concentration ranged from 2 to 8 g/L, and the MNP concentration ranged from 1 to 7.5 mg/mL. An optimized gel thickness of 3.5 mm was chosen for both plate sizes to ensure test consistency. Therefore, for the 35 mm-diameter plates, 3.4 mL of the magneto-mechanical gel solution was used, and for the 60 mm-diameter plates, 10 mL was added. Additional details on these optimization measurements, nanoparticle synthesis, and plate preparation steps are in the SI.

### 4.2 Magnetic Field Application

A static magnetic field was applied to the sample by placing a pair of permanent neodymium magnets (MIN CI Magnet Manufacturer, Amazon) on either side of the Petri dish. For the 35 mm plate, one magnet with a 3 mm thickness was attached to each side of the dish. For the 60 mm plate, four magnets were attached to each side. The field distribution across the plate was experimentally measured every 2.5 mm using a gaussmeter (Pacific Scientific Model 6010).

Finite element method modeling using COMSOL Multiphysics was performed to better understand the magnetic field gradient across the Petri dish. To allow direct comparison with the experimental results, the neodymium material N30UH was selected from the COMSOL Multiphysics library database, and the magnet strength and dimensions were set based on the experimental setup. The smallest mesh size of 40 μm was used. The recoil permeability of N30UH was 1.05, and the remanent flux density was 1.11 T. The relative permeability was set at 1 for all other areas because air, plastic, and agar are not magnetized. Both plate sizes used in the measurements were modeled.

### 4.3 Mechanical Characterization

The mechanical properties of the magneto-mechanical gel were assessed using a Dynamic Mechanical Analyzer (DMA 850, TA Instruments) using the time-oscillating method. The 35 mm-diameter Petri dishes were used to accommodate the limited space within the DMA. As described, one magnet was mounted on either side of the Petri dish, allowing for in situ actuation of the magneto-mechanical gel during the measurement. Measurements were performed with and without application of the field. The experimental setup can be found in the SI (Figure S5).

The 15 mm-diameter load cell on DMA exerted a compressive force on the gel. The test applied a displacement of 10 μm with a frequency of 0.5 Hz for a duration of 1.5 min (45 total test cycles per sample). This procedure resulted in the generation of time-dependent storage modulus and loss modulus profiles. Given the ultrasoft viscoelastic nature of the material, it was essential to introduce minimal deformation to the material during the test to ensure that the modulus readings were primarily obtained within the material’s elastic regime.^41,42^ This approach allowed for an accurate characterization of the mechanical properties of the gels.

Several control measurements were performed to ensure that the integration of the magnets into the DMA did not degrade the DMA performance. Additional experimental details, including magnet incorporation, sample parameter optimization, and test result validation, are included in the SI (Figures S6–S8).

### 4.4 Bacterial Growth

In this study, two types of media were used. For liquid culture, Luria–Bertani (LB) medium was used. LB media consisted of 25% (wt/vol) Difco LB broth, Miller (BD, USA). For solid growth substrate in plates (Figure 2), yeast extract (YE) media were used. YE media contained 0.6% (wt/vol) Bacto yeast extract (Gibco, USA), 2% (wt/vol) Bacto tryptone (Gibco, USA), and 0.5% (wt/vol) d-glucose (Amresco, USA).

All experiments used the motile strain of E. coli MG1655.^56^ Frozen glycerol stocks of the bacteria were used to inoculate 5 mL of sterile LB media and incubated at 37 °C for 18 h at 200 rpm to grow primary cultures. The cultures were then used to inoculate fresh LB broth at 1% inoculum to start secondary cultures, which were incubated at 37 °C and 200 rpm until the optical density of the culture at 600 nm reached 0.5. Cultures were then washed thrice with 1× PBS, resuspended in 5 mL of sterile 1× PBS, and used for experiments. The secondary cultures were spotted in 5 μL aliquots at the center of the 60 mm prepared Petri plates and allowed to dry for 5–10 min before incubating the plate at 37 °C for approximately 20 h. A detailed protocol can be found in the SI (Figure S9).

The biotoxicity of the nanoparticles was assessed by monitoring the growth of E. coli MG1655 populations in YE culture media without MNPs and YE culture media with 1 mg/mL of MNPs. These liquid cultures were incubated at 37 °C, at 200 rpm for 6 h. At the end of each hour, 5 μL of cultures was aliquoted and diluted to an appropriate cell density. 5 μL of each diluted culture was then spotted on YE agar plates and incubated at 37 °C overnight. The results are in the SI (Figure S10).

### 4.5 Bacterial Growth Characterization on a Magneto-Mechanical Gel Surface

The cell growth was measured in terms of colony forming units per milliliter (CFU/mL). To enable continuous monitoring of bacterial growth behavior, a Raspberry Pi-based time-lapse camera setup along with LED lights was directly integrated into the incubator. Photographs of the plates were captured at half-hour intervals throughout the entire incubation period. This data acquisition approach allowed for the cultures to be monitored without being removed from the incubator, which could introduce motion artifacts or other confounding factors into the measurements.

The images were analyzed using ImageJ, and the cumulative area occupied by the bacteria was calculated. The growth rate was calculated from these images by finding the slope of the exponential region of the growth curve. Additional details and images of the measurement setup and image analysis approach are in the Supporting Information (Figures S11 and S12).

## Supplementary Material



## Notes

Experimental details on magnetic nanoparticle synthesis, magneto-mechanical gel preparation, stability analysis, and optimization; information about magnetic field application, mechanical test setup and further material characterizations, bacterial culture protocol, bacterial growth analysis, and additional measurements of bacterial growth on magneto-mechanical gel (PDF)
