# Supplementary material for: Magnetically Tunable Hydrogel for Biofilm Control
Source: ACS Appl Bio Mater. 2025 May 19;8(6):5090–7. doi: 10.1021/acsabm.5c00409 (PMC12175159; doi:10.1021/acsabm.5c00409)
Supplement: Supplementary file 1 [file mt5c00409_si_001.pdf]

Supporting Information

Magnetically Tunable Hydrogel for Biofilm Control

*Ruojiao Sun[a], Manasi S. Gangan[b], Qiming Wang[c], James Q. Boedicker[b], Andrea Armani[*ad]*

a. Mork Family Department of Chemical Engineering and Materials Science, University of Southern California, Los Angeles, CA 90089, USA

b. Department of Physics and Astronomy, University of Southern California, Los Angeles, CA 90089, USA

c. Sonny Astani Department of Civil and Environmental Engineering, University of Southern California, Los Angeles, CA 90089, USA

d. Ellison Medical Institute, Los Angeles, CA 90064, USA

*Corresponding Author, Email: aarmani@emila.org

Table of Contents

1 Preparation of magnetomechanical gel samples and dishes S2
1.1 Magnetic nanoparticle synthesis S2
1.2 Magnetomechanical gel sample preparation S3

1.3 Stability analysis S4
1.4 Gel optimization studies S7
2 Magnetic field application S7
3 Material characterization setup S9
4 Mechanical characterization S10
5 Bacterial culture S13
6 Bacterial growth imaging and image analysis S15
7 Additional measurements for bacterial growth on magnetomechanical gel S17
8 References S18

1 Preparation of magnetomechanical gel samples and dishes

1.1 Magnetic nanoparticle synthesis

We used a coprecipitation method to synthesize all the Fe_3O_4 magnetic nanoparticles (MNP) we used in this study.[1] The synthesis procedure used is as follows.

First, measure out iron (II) chloride and iron (III) chloride in 1:2 molar ratio in a glovebox under argon. Put the salts in a three-neck reaction flask and seal all openings with rubber septa before taking it out of the glovebox and connecting it to a purged Schlenck line with a condenser. It is important there is no oxygen in the system to avoid oxidizing the iron chloride precursors.

Then add DI water to the reaction flask using a syringe needle and heat the precursor and water solution to 80 °C on a hot plate while purging the Schlenck line and the condenser. Once the solution stabilizes at 80 °C, add ammonia hydroxide dropwise to the solution while constantly stirring. The volume of the base should be of ¼ of the amount of water. After adding the base, the solution is continuously heated at 80 °C for the reaction. After 1 hour of reaction, the condenser and the Schlenk line are removed, and the reaction flask is left to cool.

We use a strong magnet and a centrifuge (3500 rpm for 15 min) to separate the synthesized nanoparticles from the solution and rinse them with water 3 times. The final dispersion is stored in sterile DI water.

The nanoparticle size and size distribution were analyzed using dynamic light scattering (DLS), and they were determined to be 124.8 nm and 88.0%, respectively. The results are shown in Figure S1.

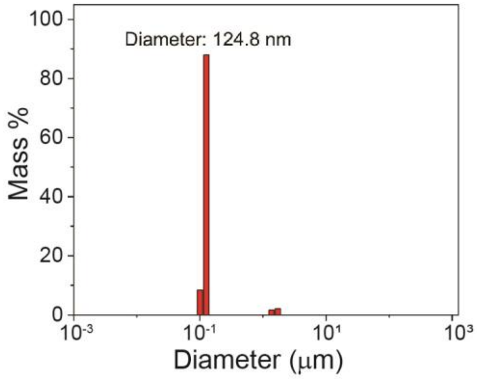

Figure S1: DLS results on the synthesized iron oxide nanoparticles used in this research.

1.2 Magnetomechanical gel sample preparation

As illustrated in Figure S2, the magnetic agar samples are prepared by combining two components: the agar-culture media solution (yeast extract media) and the magnetic nanoparticle solution. The yeast extract media (YE) consists of 0.6% (wt/vol) Yeast Extract, 2% (wt/vol) Tryptone, and 2 to 8 g/L bacterial agar.[2] The magnetic nanoparticles are synthesized in lab as in section 1.1 and stored in sterile DI water.

To make the magnetic agar sample, we weigh each part of the agar-culture media components and dissolve them in DI water. Media is then sterilized by autoclaving at 121 ºC under 15 psi for 30 mins. While the solution is approximately at 60 ºC and remains a liquid, a predetermined

amount of magnetic nanoparticle solution is added to make the gel solution a desired nanoparticle concentration. With the exception of the optimization studies described in the next section, all measurements were performed with either 5.0 mg/mL or 7.5 mg/mL nanoparticle concentrations, and 2.5 g/L agar concentration. Additionally, media is supplemented with 5% (wt/vol) glucose.

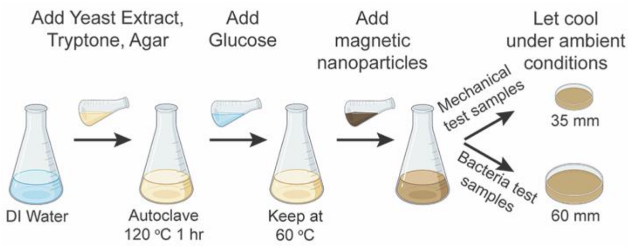

Figure S2. Schematic of magnetomechanical hydrogel preparation. Agar-based hydrogel optimized for *E. coli MG1655* growth is prepared per manufacturer instructions and prior work. Immediately before making plates, magnetic nanoparticles of varying concentrations are mixed into the solutions. In addition, plates without nanoparticles are prepared as controls.

With this magnetic gel solution, we prepare sample plates. 10 mL solution is added to 60 mm petri dishes and left to cool down and solidify at room temperature. 3.4 mL solution is added to 35 mm petri dishes and left to cool down and solidify at room temperature. The 60 mm plates are used for biological tests, i.e., to be inoculated with *E. coli MG1655*. The 35 mm plates are used for mechanical tests. In addition, sample plates with no nanoparticles were prepared as controls.

1.3 Stability analysis

The integrity and stability of the magnetomechanical gels were primarily characterized through diffusion tests and microscopic imaging. Several different tests were run using a model platform that allowed clear identification and tracking of the nanoparticle motion and diffusion.

The model system was comprised of two materials: 1) plain agar (5 g/L) and 2) magnetic nanoparticle or dye-loaded agar. To create a boundary between the two materials, the plain agar was first completely cured. Subsequently, a portion of the agar was removed, and then the hole or holes were back-filled with the nanoparticle or dye-loaded agar. For all tests, the magnetic nanoparticles were loaded at a concentration of 1 mg/mL. Additional control tests used a small molecule dye (bromophenol blue, Sigma Aldrich) at a concentration of 0.25% (wt/vol). The first series of tests investigated the porosity of the agar. Assuming minimal interactions between the pores and the nanomaterial, it is expected that the nanomaterials will diffuse into the plain agar regions to reach a thermodynamic equilibrium.

As shown in the schematics in Figure 3(a) in the main text, each agar gel had three circular regions removed and backfilled with either magnetic nanoparticle or dye. The samples were prepared in tissue culture-treated 100 mm culture dishes (Corning™, Fisher Scientific, USA) to facilitate inspection and comparison, and they were observed over a 24-hour period, a time frame comparable to that of bacterial culture experiments. As can be seen in Figure S3, the small dye molecule easily diffuses out over the 24-hour timeframe. In contrast, the nanoparticle remains confined within the original region. These images were quantified, and the total area change is plotted in Figure 4 in the main text. The data for the dye is fit to the nonlinear diffusion curve following Fick's second law relating the diffusion process with the change of area over time. The area of interest is represented as A and time is represented as t. The nonlinear fit follows a power-law model: $A(t) = A_0 + k * t^n$ where A_0 is the initial area, k is a proportionality constant, and n represents the rate of area expansion. The diffusion coefficient D is calculated by taking the derivative of A(t), which can be expressed as $\frac{dA(t)}{dt} = D(t) = n * k * t^{n-1}$. The curve fit has a R^2

of 0.98 and the value of A_0, k, and n are 202.84, 1279.87, and 0.57, respectively. The diffusion slows down as time goes on.

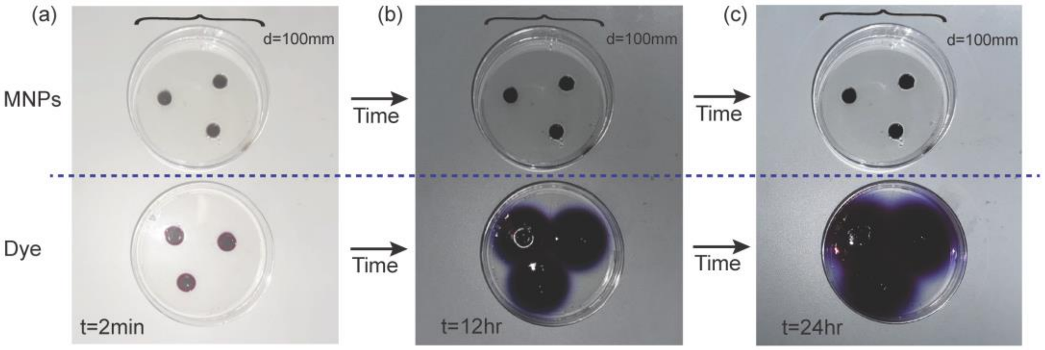

Figure S3. Comparison of the diffusion behaviors of bromophenol blue dye and magnetic nanoparticles in agar matrix over 24 hours. (a) Images taken at min 2 of the 100 mm test plates. (b) Images taken at hr 12 of the 100 mm test plates. Dyed regions started to overlap and quantification analysis was stopped at this time. (c) Images taken at hr 24 of the 100 mm test plates. MNP regions showed no diffusion activity compared to the dyed areas.

The second series of tests investigated if the continuous application of a magnetic field for 24 hours could force the nanoparticles to move into the plain agar matrix. For this series of tests, only a single region of the gel was removed, and confocal imaging was performed at different z-axis planes. This technique allows "optical sectioning" of a sample or imaging to be performed at multiple heights without physically disturbing the sample. To improve compatibility with the confocal system, we used clear TC-treated 24-well well plates (Corning™, Fisher Scientific, USA). The specific microscope used was the Operetta CLS high-content analysis system (PerkinElmer, USA). Figure 5 (b) in the main text shows the images of the magnetomechanical gel and agar gel boundaries at 3 different gel depths at 0 hours and after 24 hours of field exposure.

As can be seen, the nanoparticles remained uniformly distributed within the matrix in all three dimensions.

1.4 Gel optimization studies

As part of this work, we performed a series of optimization measurements, exploring a range of agar concentrations and a range of nanoparticle concentrations. The focus of these measurements was to maximize the magnetic response while minimizing the required concentration of nanoparticles and potential biotoxicity to the bacterial cultures.

During these exploratory studies, we co-varied the agar and the nanoparticle concentrations. Specifically, we made agar gels with 2 to 8 g/L agar concentration and varied the nanoparticle concentration from 1.0 to 7.5 mg/mL. With this magnetic gel solution, we prepared three sample plates for each agar and nanoparticle combination and magnetic field condition. 10 mL solution was added to 60 mm petri dishes and left to cool down and solidify at room temperature. 3.4 mL solution was added to 35 mm petri dishes and left to cool down and solidify at room temperature. The 60 mm plates were used for biological tests, i.e., to be inoculated with *E. coli MG1655*. The 35 mm plates were used for mechanical tests. In addition, sample plates with no nanoparticles were prepared as controls. Every experiment was done three times to ensure reproducibility of the findings, and each experiment included triplicate samples.

2 Magnetic field application

A static magnetic field was generated by positioning a pair of permanent neodymium magnets (MIN CI Magnet Manufacturer, Amazon) on opposite sides of the petri dish. For a 35 mm plate, a single 3 mm-thick magnet was affixed to each side, while for a 60 mm plate, four magnets were

used per side. The magnetic field distribution across the plate was experimentally mapped at 2.5 mm intervals using a Gaussmeter (Pacific Scientific Model 6010).

During the modeling process in COMSOL Multiphysics, a minimum mesh size of 40 μm was used to ensure high simulation accuracy. This fine mesh allowed us to capture the subtle variations in the magnetic field, especially around the magnets and in the center of the petri dish. The choice of mesh size was a trade-off between accuracy and computational efficiency – 40 μm provided a high-resolution field distribution while keeping the computational time reasonable.

For the material properties in the simulation, data of N30UH from Hard Magnetic Materials in the COMSOL Multiphysics library were used to ensure accuracy. Specifically, the recoil permeability of the N30UH magnets was 1.05, which indicates that the magnets can retain their magnetic properties after exposure to an external magnetic field. The remanent flux density was 1.11 Tesla (T), representing the magnetic flux density that the magnets can retain in the absence of an external magnetic field. Additionally, the relative permeability was set to 1 in all other regions, including air, plastic, and agar, as these materials are non-magnetic and do not significantly affect the magnetic field. This simplified the model while still maintaining the accuracy in terms of the magnetic field distribution. Both petri dish sizes used in the experimental measurements, 35 mm and 60 mm, were modeled in the simulation (Figure S4).

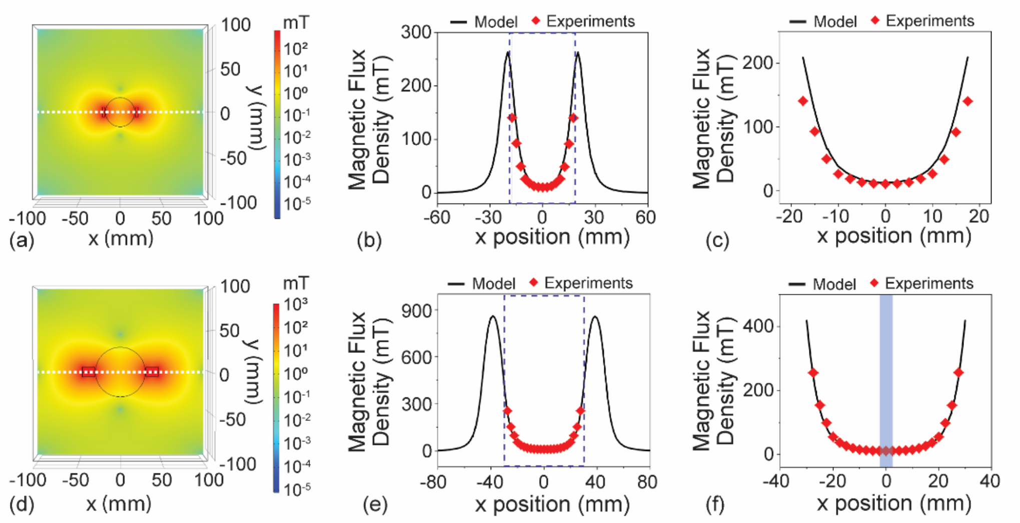

Figure S4. (a) COMSOL modeling results for 35 mm petri dish. (b) The distribution of magnetic flux density along the white dotted line at y= 0 of the 35 mm dish. The black dash lines indicate the effective bacterial growth region. (c) The distribution of magnetic flux density of the 35 mm dish within the effective bacterial growth region. (d) COMSOL modelling results for 60 mm petri dish. (e) The distribution of magnetic flux density along the white dotted line at y = 0 of the 60 mm dish. The black dash lines indicate the effective bacterial growth region. (f) The distribution of magnetic flux density of the 60 mm dish within the effective bacterial growth region. The shadowed blue region represents the inoculation spot with the diameter of 5 mm.

3 Material characterization setup

The Dynamic Mechanical Analyzer (TA Instruments DMA850), or DMA, is set on oscillation mode, where it compresses and relaxes at a frequency of 0.5 Hz with a magnitude of 10 µm. Each sample was tested under this setting for 1.5 minutes for a total of 45 cycles. The agar samples were kept in petri-dishes during testing. Due to the size limitation of the DMA sample stage, we used 35 mm petri-dishes. The load cell itself is 15 mm in diameter, and the pre-load is 0.01 N.

The key step is to provide the same magnetic field to the mechanical testing setup as the one in bacterial culture setup. However, the sample size is smaller, which means that the absolute magnetic field used should be lower. The modeling provided insight into this question, and we incorporated permanent magnets into the DMA test stage as shown in Figure S5.

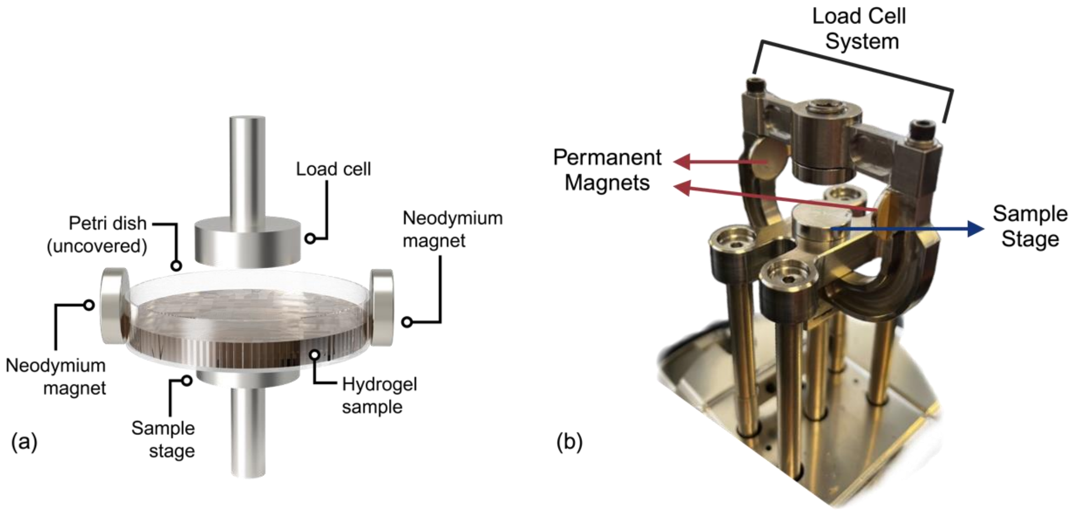

Figure S5. DMA setup with permanent magnets attached in (a) rendering and (b) actual setup.

4 <u>Mechanical characterization</u>

The mechanical characterization of agar and magnetomechanical gel started with DMA parameter optimization, sample dimension optimization, and result validation.

The optimized test parameters and settings are mentioned previously in Section 3. All mechanical test results shown below were collected under the same test conditions. Each test condition was tested in triplicates on three different occasions.

Figure S6 presents the mechanical testing results from the sample thickness trials. We found the thinnest layer that can provide an accurate, comparable to previous research, and reproducible

mechanical characterization is 3.5 mm. This layer is formed using 10 mL of gel solution in the 60mm diameter plates and 3.4 mL of gel solution in the 35 mm diameter plates.

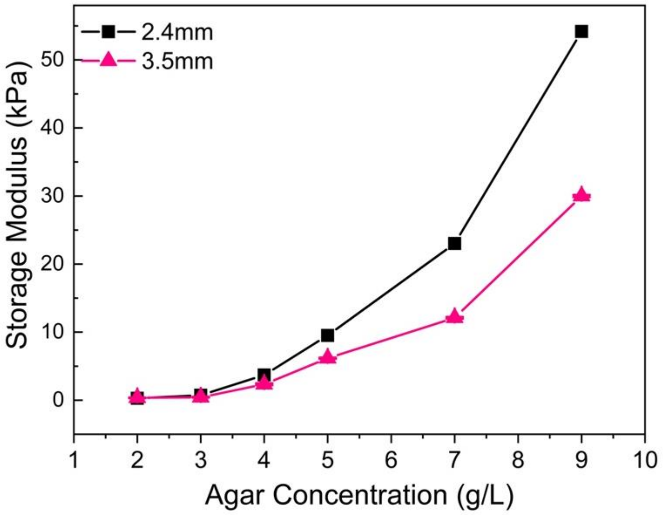

Figure S6. Agar gel thickness screening.

Figure S7 (a) and (b) show the direct output from the DMA for the agar concentration screen. The samples were made with agar concentrations ranging from 2 to 8 g/L. These samples were run for 5 minutes, or 150 cycles, to demonstrate the test parameters do not inflict damage to samples during testing. As we can see, the storage modulus readings of tested agar samples are stable and consistent over the duration of this testing time. The loss modulus of these samples is less than 20% of their storage modulus. This material behavior during testing indicates that the viscoelastic nature of the material is not interfering with the measurement accuracy and reproducibility. Figure S7 (c) is a comprehensive summary of the storage and loss modulus of these samples with different agar concentrations. Each data point includes test results from a triplicate of samples.

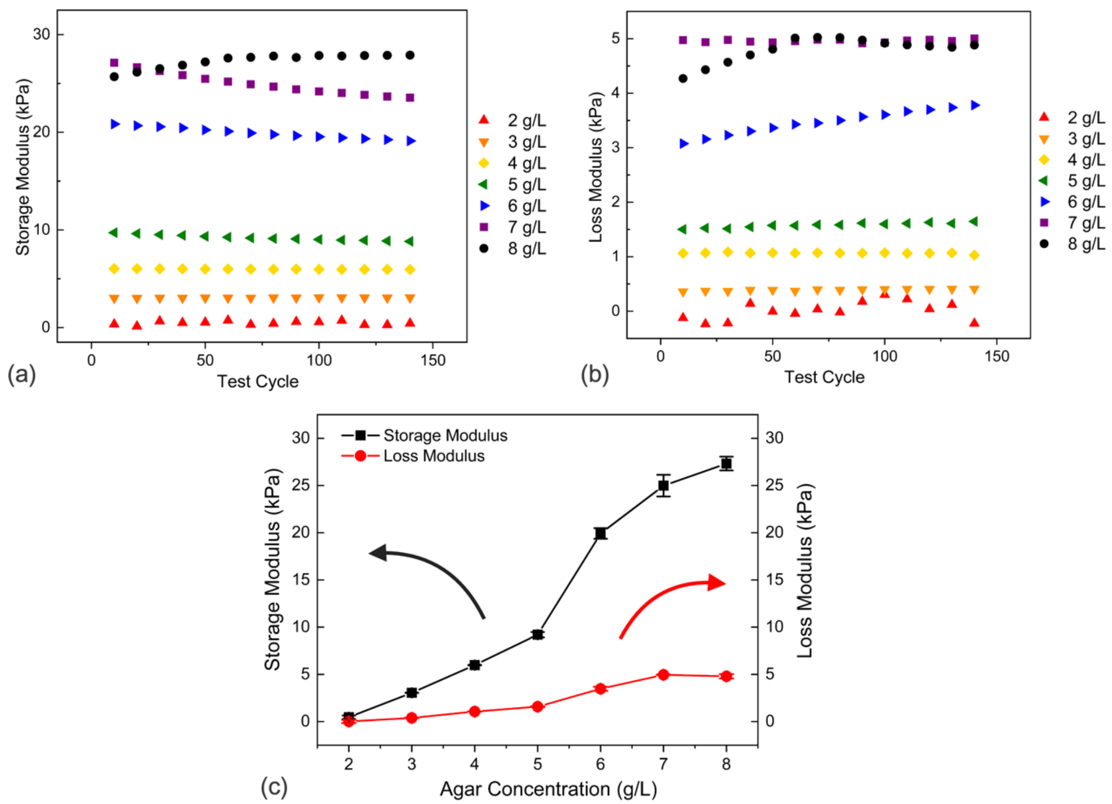

Figure S7. DMA results on the storage (a) and loss modulus (b) profile of gels with different agar concentrations. (c) Storage and loss modulus of agar gel as agar concentration increases.

An important control test is to ensure that the presence of the magnetic field is not interfering with the DMA reading. As a control, we tested agar samples with no nanoparticles to compare the modulus measurements with a magnetic field applied and not applied. The DMA readings were not affected by the magnetic field (Figure S8). Therefore, we used this setup for agar screening with different field conditions and nanoparticle concentrations.

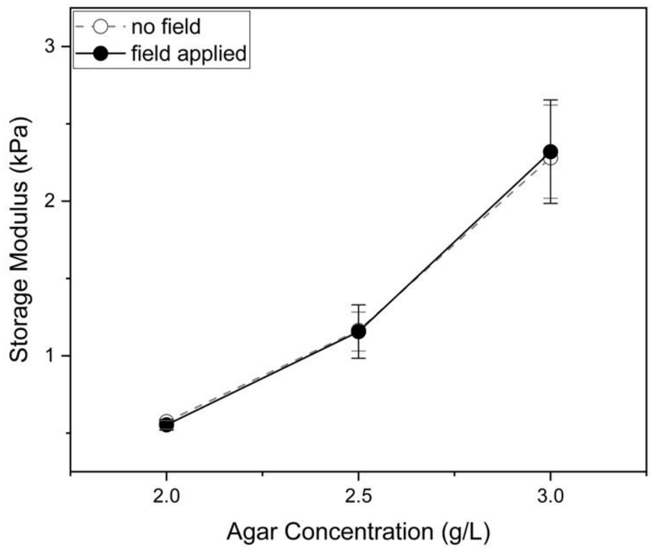

Figure S8. DMA storage modulus reading of agar samples without nanoparticles tested with magnetic field applied and not applied. Test were done in triplicates.

5 Bacterial culture

As shown in Figure S9, the bacteria were cultured from frozen glycerol stocks of a motile strain of *E. coli MG1655*. [3] The primary culture consists of 5 mL of LB media and was incubated at 37 ºC and 200 rpm for 18 hours. Primary cultures were then used to inoculate fresh LB at 1% inoculum to start secondary cultures, which were incubated at 37 °C at 200 rpm for approximately 2 hours until the culture reaches the exponential phase and optical density (OD) is approximately 0.5. The OD of the secondary culture is measured using a spectrophotometer (Spectronic™ 200, Thermo Scientific). Cultures were then washed thrice with sterile 1X PBS, resuspended in 5 mL sterile 1X PBS, diluted to OD = 0.5, and made ready to inoculate on the magnetic agar gel plates.

5 μL of the secondary culture with 0.5 OD was inoculated at the center of each 60 mm agar gel plates. The plates were left undisturbed for 5-10 minutes to allow the inoculation spot to dry. The plates were then put into an incubator at 37 °C for 20 hours.

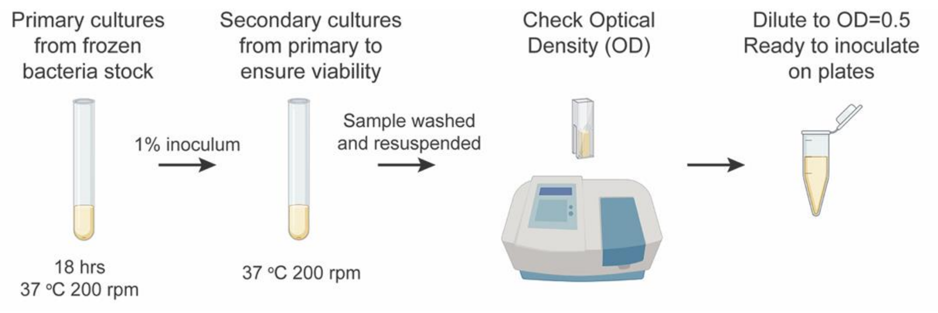

Figure S9. Schematic of bacteria preparation.

To assess the biotoxicity of magnetomechanical gel on *E. coli* populations, secondary cultures of *E. coli* were grown in Yeast Extract (YE) growth media containing 1 mg/ml of nanoparticles in triplicate. Populations were incubated at 37 °C under shaking conditions (200 rpm) for 6 hrs. Viability of the culture was tested every hour in terms of colony forming units (CFU). This was done by withdrawing 100 μL of cultures at one-hour intervals and diluting them to appropriate cell densities. 5 μL of the last three dilutions were then spotted on fresh agar plates. The plates were then incubated approximately for 12 hours at 37 °C. Full grown and well segregated colonies were counted and corresponding dilution factor was used to back-calculate CFU/mL.

For the control experiments, the calculated doubling time for *E. coli MG1655* populations in YE media was found to be 31 ± 1 min. It is comparable to previously reported values.[4] Upon addition of 1 mg/mL of magnetic nanoparticles in the YE media, the doubling time showed slight increases to 33 ± 2 min, respectively (Figure S10), indicating that the addition of the magnetic nanoparticle in the cell environment has no diminishing influence on the bacterial growth.

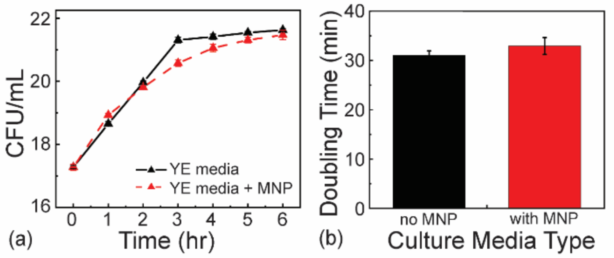

Figure S10. Assessment of biotoxicity of magnetic nanoparticles. *E. coli MG1655* cells were grown in Yeast Extract (YE) broth. (a) Their recorded growth over 6 hrs in the presence of 1 mg/mL of magnetic nanoparticles is shown with dash lines, while control experiments are shown with solid lines. (b) The bar graph represents respective doubling time of an individual population in YE growth media.

6 <u>Bacterial growth imaging and image analysis</u>

We implemented a Raspberry-Pi driven camera setup to monitor the bacteria growth and motion during the incubation period. The camera setup consists of LED light bars (SAMTIAN 84 pcs LED, Amazon), a 4K resolution webcam (Logitech Brio 4K Webcam), and a Raspberry-Pi board (Model 3B, Raspberry Pi). We used a black background underneath our sample plates to create a uniform background and increase the contrast to aid in the subsequent image analysis. The Raspberry-Pi ran a code where an image is taken every 30 minutes and uploaded to the cloud. The LED light bar is kept on during the incubation period (Figure S11).

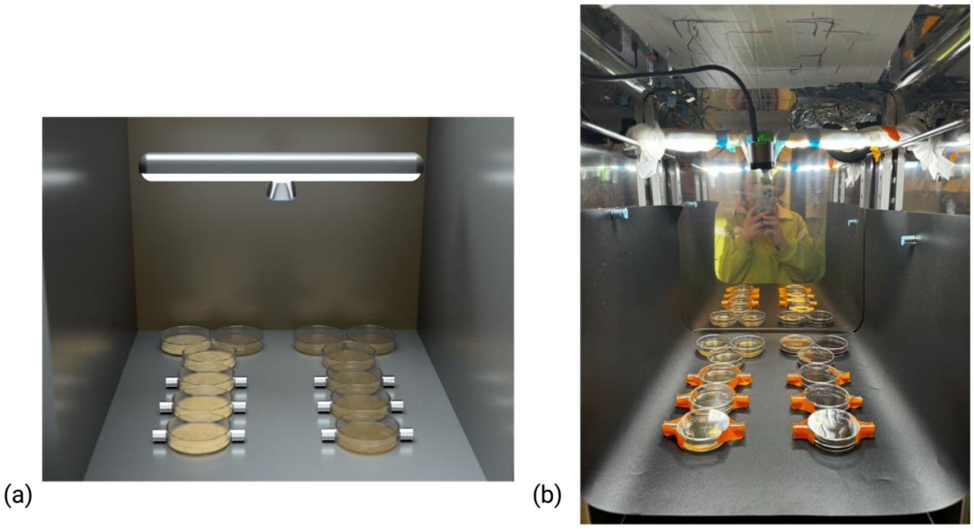

Figure S11. Camera set-up inside the incubator in (a) rendering and (b) actual setup.

The captured images can be edited into a time-lapse video, or they can be analyzed individually. We used ImageJ to change the images to greyscale (8-bit) and adjust the brightness and contrast. Then we applied the Spectrum filter to make the bacteria growth pattern stand out more from the background and used the Freehand Selection tool to outline the bacteria pattern area. This process is shown stepwise for a single image in Figure S12. Due to the irregularity of the bacteria growth pattern, we decided to use area change instead of the radius change in the rate analysis.

With the Set Scale and Measure tools in ImageJ, we could determine the area of the selected pattern area. Using the time stamp on each image, we calculated the biofilm expansion rate in mm^2/hr.

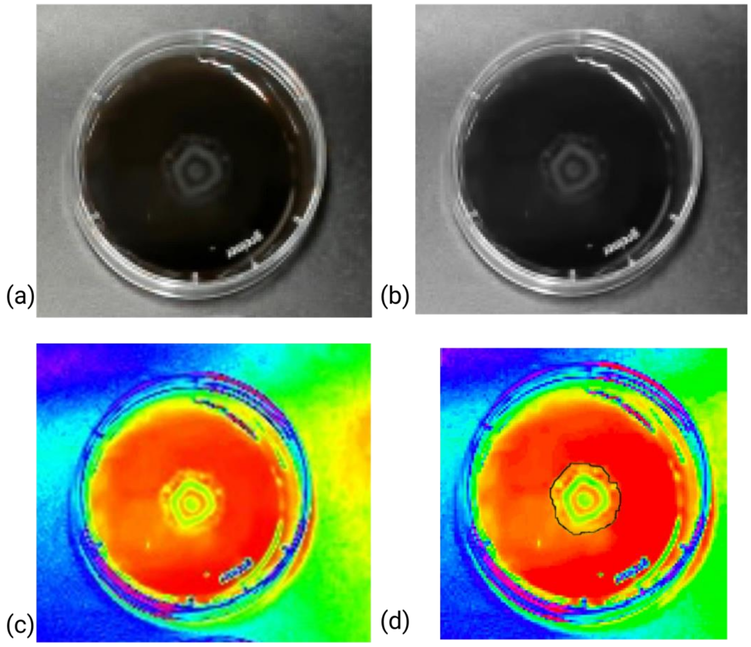

Figure S12. (a) Color image from the Raspberry-Pi webcam setup, (b) Same image in greyscale with contrast and brightness adjusted, (c) Same image after Spectrum filter applied, (d) Same image with bacteria growth pattern area selected.

7 Additional measurements for bacterial growth on magnetomechanical gel

Three samples were made for each testing condition, and the same experiment was repeated at three different occasions. Figure S13 shows biological examples of the magnetomechanical gel of 2.5 g/L agar and 5 mg/mL or 7.5 mg/mL nanoparticles when cultured with and without magnetic field.

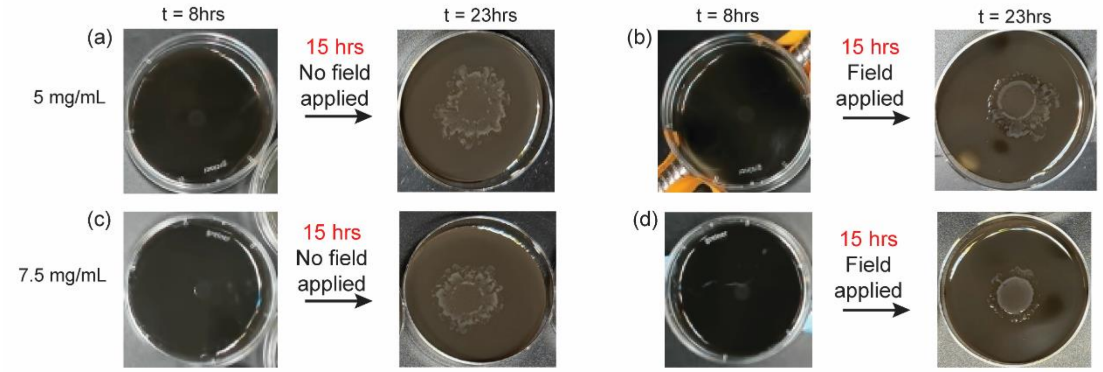

Figure S13. Biological test results on magnetomechanical gels of 2.5 g/L agar with 5 mg/mL and 7.5 mg/mL nanoparticle concentrations with magnetic field on and off. (a) Magnetomechanical gels with 5 mg/mL nanoparticle cultured without applied magnetic field. (b) Magnetomechanical gels with 5 mg/mL nanoparticle cultured with magnetic field applied. (c) Magnetomechanical gels with 7.5 mg/mL nanoparticle cultured without applied magnetic field. (d) Magnetomechanical gels with 7.5 mg/mL nanoparticle cultured with magnetic field applied. The color differences are due to different camera and lightings at hr 8 and hr 23. At hr 8, the images were taken through the 4K web camera in the incubator and at hr 23, the images were taken with a mobile phone in a photo box with stronger lighting as endpoints.

8 References

(1) Wu, W.; He, Q.; Jiang, C. Magnetic Iron Oxide Nanoparticles: Synthesis and Surface Functionalization Strategies. *Nanoscale Res. Lett.* **2008**, *3* (11), 397–415. https://doi.org/10.1007/s11671-008-9174-9.

(2) Burkart, M.; Toguchi, A.; Harshey, R. M. The Chemotaxis System, but Not Chemotaxis, Is Essential for Swarming Motility in Escherichia Coli. *Proc. Natl. Acad. Sci.* **1998**, *95* (5), 2568–2573. https://doi.org/10.1073/pnas.95.5.2568.

(3) Tran, F.; Boedicker, J. Q. Plasmid Characteristics Modulate the Propensity of Gene Exchange in Bacterial Vesicles. *J. Bacteriol.* **2019**, *201* (7), e00430-18. https://doi.org/10.1128/JB.00430-18.

(4) Gangan, M. S.; Athale, C. A. Threshold Effect of Growth Rate on Population Variability of Escherichia Coli Cell Lengths. *R. Soc. Open Sci.* **2017**, *4* (2), 160417. https://doi.org/10.1098/rsos.160417.
